# Childhood Sjögren’s Disease: A Literature Review of an Underrecognized Autoimmune Entity in Pediatric Rheumatology

**DOI:** 10.7759/cureus.98894

**Published:** 2025-12-10

**Authors:** Uziel Márquez Romero, Andrés Sebastián Estrella López, Juan Carlos Panchi Jima, Esthela Carolina Hidalgo Tapia, Eduardo Andrés Callejas Torres, Jennifer Katherin Suarez Piñeros

**Affiliations:** 1 Intensive Care - Emergency, Hospital de Alta Especialidad de Veracruz, Veracruz, MEX; 2 Public Health, NeoScientia Consulting Group, Quito, ECU; 3 Primary Health Care, Institut Català de la Salut, Vic, ESP; 4 Nursing, Universidad de Cuenca, Cuenca, ECU; 5 Ophthalmology, Sociedad Médica Clínica Maicao, Maicao, COL; 6 Pediatrics and Neonatology, Hospital San Rafael de Alajuela, Caja Costarricense del Seguro Social, Alajuela, CRI

**Keywords:** childhood, pediatric rheumatology, pediatrics, sjögren’s disease, underrecognized autoimmune disease

## Abstract

Childhood Sjögren’s disease (cSjD) is a rare and poorly recognized systemic autoimmune disease whose clinical presentation is distinctly different from the adult form of the disease. The present synthesis is an overview of the existing literature that points to a heterogeneous disease profile, meaning recurrent parotitis and extraglandular manifestations rather than typical sicca symptoms in adults (dry eyes and mouth). One challenge in pediatric rheumatology is that not all diagnostic criteria developed in adults, including the American College of Rheumatology/European Alliance of Associations for Rheumatology 2016 criteria, which have low sensitivity in children, can be applied, resulting in considerable delays in diagnosis. Research has established that a significant proportion of children who are identified with cSjD through expert opinion do not fit the standards. Major clinical signs among children consist of frequent parotitis, arthralgia, lymphadenopathy, and a broad spectrum of dysfunction in the hematological, renal, hepatic, and central nervous systems. Life-threatening complications are infrequent but include interstitial lung disease, diffuse alveolar hemorrhage, and B-cell lymphoma. The gap in diagnosis has been addressed by existing studies that aimed at non-invasive measures and novel classification systems. Salivary gland ultrasonography and ultra-high-frequency ultrasonography are emerging tools to assess the inflammation of the glands. New, empirical methods, including the Florida Scoring System, and systematic clinical models, including the three-pathway diagnostic algorithm, have been designed to offer a more precise and standard cSjD assessment. The diagnostic algorithms need to be proven by further research. The management of cSjD lacks a standard practice and is strongly dependent on individual practitioners, who often base it on adult practice. The management is specific to the clinical manifestations, with mild symptoms treated with non-steroidal anti-inflammatory drugs and hydroxychloroquine, and moderate or severe systemic disease and complications treated with corticosteroids, conventional disease-modifying antirheumatic drugs, and biologics such as rituximab. This review highlighted the constitutional symptoms, such as rash, fever, and joint pain (arthralgia), which are common and often present alongside glandular inflammation. Neurological involvement, including psychiatric symptoms, may also occur, further complicating diagnosis and management. There is a very low-quality evidence base on these treatments, with case reports and small series as the main components, underlining the urgent need for collaboration, high-quality research, and creation of pediatric-specific diagnostic criteria as well as treatment guidelines.

## Introduction and background

Sjögren’s disease (SD) is a systemic autoimmune disease that causes chronic lacrimal and salivary gland inflammation and dryness [1]. Patients usually have dryness, fatigue, and diffuse pain. Peripheral neuropathies, arthritis, interstitial pneumonitis, nephritis, and skin involvement affect 30-40% of patients [2]. With a female-to-male ratio over 10:1, SD mostly affects women. The majority of cases are noted in peri- or postmenopausal women, but it can also affect children and elderly individuals. Population-based research estimates SD presence at 4-5 cases per 10,000 individuals [3].

Clinical presentation and long-term prognosis of SD in children are less well defined [4]. Childhood-onset Sjögren’s disease (cSjD) occurs before 18 years of age. cSjD is poorly characterized and likely underdiagnosed [5], despite recent improvements in defining the illness profile in children [6]. Despite different pathophysiology, IgG4-related disease in infants sometimes appears as ocular disease and shares clinical characteristics [7].

As cSjD has no gold-standard diagnostic tool, diagnosis is predicated on expert clinical judgment, opinion, clinical history and examination, functional exocrine gland testing, and serological and histological evidence [8]. Children have traditionally been diagnosed with primary, but recent investigations have shown that their clinical symptoms are diverse and difficult to map against adult classification criteria [5]. Specialists suggest classifying Sjögren’s syndrome (SS) as SD rather than primary or secondary to another autoimmune condition, as this does not accurately describe the illness etiology [9]. To prevent disputes, we use SD with childhood onset instead of primary SS in children in this article.

Due to a paucity of good-quality evidence for treatment success in cSjD [10], therapeutic options for children and adults are similar and are reviewed. This review identifies and analyzes articles on pathology, mechanism, and pharmaceutical therapies for cSjD, focusing on clinical indication and efficacy. The key therapeutic tendencies in cSjD treatment approaches are also discussed to determine the challenges and future directions.

## Review

Methodology

**Figure 1:**
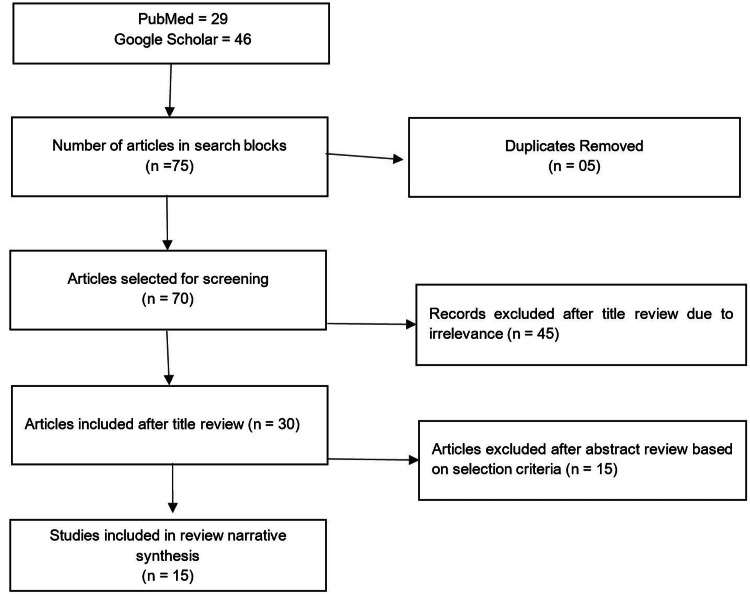
Flowchart showing the selection of prospective and observational studies for the narrative review.

This narrative literature review was conducted using relevant topic-specific keywords such as “Sjögren's Disease (SjD),” “Childhood Sjögren’s Disease (cSjD),” “Autoimmune Entity,” “Underrecognized Autoimmune Entity,” “Glandular involvement,” “Diagnostic approach,” “Mechanism of Disease,” and “Pediatric Rheumatology.” Boolean operators were used to incorporate the keywords and search on PubMed and Google Scholar, focusing on full-text open-access articles from January 1, 2015, to October 31, 2025. The literature search was limited to peer-reviewed literature in the English language. Data extraction of characteristics from studies included the author, year, major cohorts, definition and clinical features, therapeutic management, risk factors, diagnosis capability, novel approaches, and challenges in screening. The literature review focused on the current literature landscape from the last 10 years. Cohort and observational studies were selected while screening for articles to be included in the narrative review (Figure 1).

Narrative synthesis

Pathophysiology and Mechanism of Disease

SD is fundamentally a chronic, systemic autoimmune disease characterized by an immune-mediated attack on the exocrine glands, leading to inflammation and subsequent dysfunction or destruction. The hallmark histological feature is the presence of a chronic lymphocytic infiltrate, known as focal lymphocytic sialadenitis, observed in labial minor salivary gland biopsies [11]. The underlying autoimmune process involves abnormal B-cell and T-cell responses directed against autoantigens such as anti-Ro/SSA and anti-La/SSB. Lymphocytic infiltration and autoantibody production are viewed as early features in the immunopathogenesis that precede subsequent gland dysfunction and end-organ damage. Hence, children who lack full glandular function may experience an earlier stage of disease development [12].

Immunological Mechanisms

An essential peculiarity of the autoimmune process is the autoantibody development. The different serological markers include antinuclear antibodies (ANAs), anti-Ro/SSA/anti-La/anti-SSB antibodies, and rheumatoid factor (RF) [11]. The presence of such autoantibodies suggests that the pathogenesis could have occurred several years before the clinical presentation [13]. There is also likely a sign of systemic inflammation and B-cell activation manifesting as increased erythrocyte sedimentation rate (ESR), increased C-reactive protein (CRP), and hypergammaglobulinemia. The high level of IgG is typical and believed to indicate more B-cell activity. Higher CRP is much more common in patients referred with parotitis [14].

The immune mechanisms manifest differently in cSjD, often focusing on glandular inflammation and extraglandular symptoms. For instance, abnormal immunity involving IgA production may contribute to SD, as plasma cells dominantly produce IgA in the salivary glands of SD patients [15]. Involvement in extraglandular manifestations often suggests specific underlying immunological activity, such as interstitial lymphocytic inflammation in the lung (e.g., nonspecific interstitial pneumonia or lymphoid interstitial pneumonia patterns), which has been documented based on lung wedge biopsies showing interstitial lymphocytic inflammation [16], or tubulointerstitial damage in the kidney associated with lymphocytic infiltrates in the tissue. The pathogenic mechanisms underlying neurological involvement include direct infiltration of the central nervous system (CNS) by mononuclear cells, vascular injury related to the presence of antineuronal and anti-Ro antibodies, and ischemia secondary to small-vessel vasculitis [17]. The progression to extranodal marginal zone B-cell lymphoma (EMZL) is also believed to be triggered by chronic immune stimulation within the context of a pre-existing inflammatory response [15].

Genetic and Environmental Causes of cSjD

cSjD in children depends on genetic predisposition as well as environmental provocation, which is unlikely to be clear-cut despite the fact that the definite etiology is not clearly explained. On the genetic and familial side, there is also an indication that the inherited factor is also strong, as manifested by a common occurrence of autoimmunity within the families of the affected children. According to one study conducted among children presenting with enlargement of salivary glands, 70% showed a family history of several autoimmune diseases, and 20% had a family history where cSjD was found. Moreover, the existence of cSjD in the family was highly correlated with a clear cSjD diagnosis in a patient, with the influence of meeting the requirements of classification [11]. Certain genetic markers are linked to the condition, including the correlation between HLA-Dw3 and HLA-B8 and SS [13]. Genetic syndromes can also co-occur or confound with the disease, such as congenital syndromes that can induce sicca symptoms, such as lacrimo-auriculo-dento-digital syndrome, which is caused by a developmental abnormality of the salivary glands [18]. In one case, a patient diagnosed with cSjD was reported to have the chromosomal (XXX) anomaly 47 [19]. Concerning environmental and infectious factors, there is the involvement of viruses and chronic inflammation. cSjD was reported after infectious mononucleosis [13]. Broadly, autoimmune diseases or chronic infections are known to result in chronic immune stimulation, which can be the cause of downstream complications such as EMZL in theory. Although it is partially substantiated by evidence of a relationship with SD, the clear causal association remains uncertain and needs additional research [15]. Chronic sialadenitis is a typical manifestation of cSjD, which is also included in the differential diagnosis of other infectious diseases such as hepatitis C and tuberculosis [18]. Moreover, recurrent salivary gland enlargement was also associated with environmental allergy, more prevalent in 23% of the children studied, with some in this cohort also manifesting certain types of immune deficiency, such as IgA immunodeficiency [11]. These results imply that genetic vulnerability and environmental exposure or immune perturbations are taken together as factors associated with the cSjD development.

Comparison of the Mechanism of Pediatric Autoimmune Disease With Adults

SD is an autoimmune and systemic chronic disease whose symptoms and pathogenesis vary largely when comparing cSjD and adult-onset manifestations, which are mostly due to differences in the disease progression phase and the body organs affected by both active and previous inflammation [8,20]. Several pediatric cohorts have summarized the key clinical features of cSjD (Table 1), which help in understanding its clinical presentation. A major difference between cSjD and adult SD lies in glandular involvement. Adult SD is typically marked by the development of sicca symptoms, such as xerostomia (dry mouth) and xerophthalmia (dry eyes) [6]. However, in children, these symptoms are less common at the time of initial diagnosis and tend to develop later [21]. Instead, recurrent or persistent parotitis (salivary gland enlargement) is the most frequent presenting symptom in children [22]. This parotitis is strongly associated with a younger age at diagnosis [23]. Overall, the clinical presentation of cSjD exhibits a broad spectrum that clearly distinguishes it from adult SD.

**Table 1:** Clinical features of cSjD reported in key pediatric cohorts. CNS = central nervous system; SSA/Ro = Sjögren’s syndrome antigen A (also known as Ro); SSB/La = Sjögren’s syndrome antigen B (also known as La); ANA = antinuclear antibody; cSjD = childhood Sjögren’s disease

Clinical features	Marino et al. 2020 (n = 240) [13]	Basiaga et al. 2021 (n = 300) [8]	Ramos-Casals et al. 2021 (n = 158) [6]	Gong et al. 2023 (n = 39) [19]
Median age at diagnosis	10 years (onset)	12 years	14.2 years	10.9 years
Female predominance	9 (79.6%)	249 (83%)	136 (86.1%)	31 (79.5%)
Parotitis	8 (65%)	147 (49%)	100 (63%) (enlargement)	10 (25.6%)
Dry mouth	7 (59%)	156 (52%)	126 (80%)	9 (24.1%) (dry mouth/eyes)
Dry eyes	6 (56%)	144 (48%)	111 (70%)	8 (23.1%) (dry mouth/eyes)
Arthralgia/Arthritis	10 (81%)	161 (54%) (arthralgia), 72 (24%) (arthritis)	42 (26.5%) (articular domain)	7 (17.9%) (arthralgia)
Rash/Cutaneous	8 (65%)	27 (9%) (vasculitis)	19 (12.3%) (cutaneous domain)	20 (51.3%)
Renal involvement	7 (59%)	27 (9%) (any renal)	7 (4.5%) (renal domain)	8 (20.5%)
CNS involvement	11 (85%)	33 (11%) (any neurological)	1 (0.6%) (CNS domain)	1 (2.6%)
Anti-SSA/Ro positive	11 (85%)	222 (74%)	131 (82.7%)	39 (100%)
Anti-SSB/La positive	7 (59%)	135 (45%)	98 (61.9%)	18 (46.1%)
ANA positive	11 (85%)	264 (88%)	143 (90.3%)	37 (94.9%)

This clinical divergence among children and adults is theorized to reflect different stages of immunopathogenesis. The underlying immune mechanisms involve lymphocytic infiltration and autoantibody production, which are considered early features preceding subsequent gland dysfunction and end-organ damage [12]. Because children may not have accumulated long-standing glandular damage, they may represent an early stage in the disease spectrum [6]. Consequently, adult classification criteria relying on specific objective evidence of glandular dysfunction (such as decreased tear or saliva production) are often not sensitive for diagnosing SD in children, as this dysfunction is less evident in pediatric patients [18]. For instance, the traditional cutoff for focal sialadenitis (a focus score ≥1 focus/4 mm²) may be inappropriate for children, suggesting that milder signs of inflammation (a focus score >0) might suffice to support a diagnosis in this younger, earlier-stage population [8,12]. Furthermore, the glandular tissue in pediatric patients is less likely to exhibit fibrosis and fatty infiltration compared to adults with long-standing disease [20].

Differences in Systemic Activity Patterns

While cSjD is recognized for its prominent glandular inflammation, it is also highly systemic. cSjD shows the highest mean European League Against Rheumatism Sjögren's Syndrome Disease Activity Index (ESSDAI) score and the highest frequencies of positive autoantibodies compared to all adult age subsets, indicating robust systemic activity early in the disease course [6]. However, the specific patterns of extraglandular manifestations differ based on age.

Increased activity in young patients: A younger age at diagnosis is associated with a greater risk of active disease presentation in domains such as constitutional, lymphadenopathy, glandular, cutaneous, and hematological involvement. Skin involvement, including rash or purpura, was the main clinical manifestation in one Chinese pediatric cohort (51.3%) [19].

Decreased activity in young patients: Pediatric patients have a statistically decreased risk of presenting activity in the pulmonary and neurological domains compared to older adult cohorts [6]. Nevertheless, serious organ involvement, such as neurological disorders, diffuse alveolar hemorrhage, and interstitial lung disease, still occurs in pediatric cases, often presenting atypically [22]. For example, neurological involvement in adult SD is relatively common, whereas CNS and renal damage are described as uncommon in pediatric cases [17]. Hence, cSjD is an immune-manifested disease that presents with glandular inflammation (parotitis), but not glandular damage (sicca symptoms), typically with cutaneous and hematological systems involvement, and showing a general high serological and systemic response. The presence of these inherent differences leads to the continuous work on creating the pediatric-based diagnostic and classification criteria because the adult criteria do not reflect the characteristic disease phenomenon in the earliest stages [8,18,20].

Clinical presentation and clinical management consequences of these differences

The differences in how cSjD and adult SD manifest have significant implications for their clinical presentation, diagnosis, and treatment. One of the key differences lies in the age of onset and the progression of the disease. While adults often experience an earlier stage of disease accumulation, children tend to develop the disease at a later stage, which affects how the disease appears and progresses in each group [8,12].

Symptom Spectrum and Preeminence

The disease traditionally has sicca symptoms (dry eyes and dry mouth) in the adult case as a result of long-term destruction of exocrine glands and is observed in more than 95% of patients [6]. On the other hand, cSjD tends to manifest itself in a non-traditional manner, including glandular inflammation over dysfunction and extraglandular prominence [19]. Recurrent or persistent parotitis (glandular enlargement) peaks the clinical manifestation of glandular inflammation over dysfunction in the pediatric population, with an overall reported incidence of 49% to 68% in children [22,23]. This is as opposed to the reduced incidence of classical sicca symptoms at first diagnosis in children that occur later in the disease progression [20,22]. Children with cSjD often exhibit EGMs. In one study, 51.3% of patients presented with rash or purpura [19], 54% with arthralgia [8], and others with fever and hematological involvement [6]. Constitutional symptoms, lymphadenopathy, glandular issues, cutaneous manifestations, and hematological activity are more commonly observed in younger patients. This clinical heterogeneity complicates diagnosis, making it crucial to maintain a high index of suspicion [18].

Diagnostic Challenges

The most dire implication is that adult criteria of classification (e.g., the 2016 American College of Rheumatology (ACR)/European Alliance of Associations for Rheumatology (EULAR) criteria) fail to properly classify cSjD because of poor sensitivity and the use of dysfunction markers, which underlines the necessity of special tools [18,20]. A large majority of children (77% in a multinational cohort) with SD did not fulfill the 2016 ACR/EULAR classification criteria, suggesting low sensitivity [20,22]. Only 36% of the children had undergone all necessary tests and yet satisfied the criteria [8]. In adults, the criteria are overly dependent on objective evidence of dysfunction of the glands (low quantity of tears/saliva). Glandular dysfunction cannot always be evident in children because it takes time (damage accrual) to manifest [12,20]. However, objective methods such as the Schirmer test and unstimulated whole salivary flow do not have many pediatric-specific normative values or are hard to perform because of the inability of children to be cooperative [20,24].

There is a need for special instruments because, due to a lack of appropriate criteria, one resorts to clinical judgment with reference to adult criteria [23]. This has led to the establishment of other non-invasive methods, such as salivary gland ultrasonography, and new methods such as ultra-high-frequency ultrasound of the labial glands, which aim to detect early inflammation before dysfunction in the gland becomes apparent [24].

Prognostic Differences

Although long-term prognosis remains poorly understood in children, the prognostic features found in cSjD resemble the features observed in cases of worse outcome in adults, including higher systemic activity, malignancy risk, and progression to overlap diseases. In cSjD, the mean ESSDAI score and the positive autoantibody frequencies are the best when compared to all other age subsets of the disease, implying a vigorous systemic response during disease progression [6]. Characteristics common to cSjD (young age at diagnosis and glandular swelling) are recognized risk factors of malignancy (especially malignancy-associated tuberculosis lymphoma) in adults [23]. There is no known absolute risk in children, but malignancies, mostly mucosa-associated lymphoid tissue lymphoma, have been reported previously in cSjD patients that are fatal in some cases [15]. This requires close follow-up and progression toward lymphoma with the frequent use of tools such as salivary gland ultrasonography [18]. One of them is the reported transition of cSjD progression to other systemic autoimmune diseases. Cases of pediatric patients who were first diagnosed with SD but subsequently experienced systemic lupus erythematosus (SLE) led to the recommendation of close and protracted consideration of SLE in patients with cSjD [19,25].

Implications for Therapeutic Management

Management strategies for cSjD often rely on extrapolation from adult studies due to the rarity of the disease and the lack of pediatric-specific trials, validated outcome measures, or FDA-approved treatments. The treatments of childhood-onset SjD are presented in Table 2.

**Table 2:** Therapeutic management derived from a systematic review of cSjD. cSjD = childhood Sjögren’s disease; NSAIDs = non-steroidal anti-inflammatory drugs; cDMARDs = conventional disease-modifying antirheumatic drugs; HCQ = hydroxychloroquine; MTX = methotrexate; AZA = azathioprine; MMF = mycophenolate mofetil; CYC = cyclophosphamide; RTX = rituximab; TNF = tumor necrosis factor; and IL-6 = interleukin-6; JIA = juvenile idiopathic arthritis; SLE = systemic lupus erythematosus; TIN = tubulointerstitial nephritis; NMOSD = neuromyelitis optica spectrum disorder; MALT = mucosa-associated lymphoid tissue; PH = pulmonary hypertension; - = not applicable The findings reported in the table were derived from the study by Doolan et al. (2022) [10].

Therapy class	Medications	Agents	Primary clinical indications in cSjD	Comments/Notes
-	Topical/Symptomatic	Artificial tears/saliva, pilocarpine, bromhexine	Sicca symptoms (dry eyes, dry mouth), and prevention of dental caries	Generally, it provides symptomatic relief. Pilocarpine showed clinical benefit in improving xerostomia
-	NSAIDs	Ibuprofen, naproxen, etc.	Arthritis, arthralgia, and fever	Used for milder musculoskeletal symptoms
-	Corticosteroids	Prednisone, methylprednisolone (oral, IV)	Severe systemic manifestations affecting the CNS, renal, and pulmonary systems; parotitis; and arthritis	Commonly prescribed (52% of children). Effective for short-term treatment because long-term use has reported side effects, with limited evidence
Conventional DMARDs	Antimalarials	HCQ	Parotid swelling, arthralgia, fatigue, skin disease, and maintenance therapy	Most frequently prescribed cDMARD (34% of children). A favorable response was reported in 39% of cases, often in combination with other therapies
	Other cDMARDs	MTX	Inflammatory arthritis, arthralgia, and purpura	Prescribed in ~6% of children. Associated with clinical benefit where reported
		AZA	Overlapping JIA/SLE, autoimmune hepatitis, and TIN	Rarely used (~2% of children)
		MMF	Severe renal disease (TIN), NMOSD, maintenance therapy for psychiatric or pulmonary manifestations	Used in selected severe cases with moderate response
		CYC	Organ-threatening complications: TIN, NMOSD, severe isolated PH, and CNS involvement	Reserved for severe, life-threatening disease with evidence of significant improvement
Biologic DMARDs	B-cell depletion	RTX	MALT lymphoma, refractory systemic disease, NMOSD, severe psychiatric symptoms, cryoglobulinemia	Reserved for severe cases. Achieved remission in MALT lymphoma and improved psychiatric symptoms
	Anti-TNF	Etanercept, infliximab	Inflammatory arthritis (often with overlapping JIA)	Used in a small number of cases with reported clinical benefit for arthritis
	Other biologics	Tocilizumab (IL-6 inhibitor)	Refractory neurological manifestations (e.g., NMOSD)	The successful control of neurological relapses after RTX failure

Glandular Management

The significance of recurrent parotitis among children predetermines special managerial strategies, i.e., sialendoscopy and objective monitoring. Sialendoscopy, frequently performed with corticosteroid irrigation (e.g., triamcinolone), is gaining acceptance as a treatment of recurrent parotitis in children, but is not mainstream care in adult guidelines. The direct visualization and treatment of ductal pathologies is made possible by the use of sialendoscopy [20]. This would be more successful, possibly, with children, in whom the glandular tissue would not have the same degree of fibrosis and fatty infiltration and inflammation that is noted in the long-lasting disease. For objective detection, salivary gland ultrasound is applied to test the effectiveness of interventions and measure the inflammatory dynamics. It assists clinicians in monitoring the progress where no pediatric measures of outcomes have been validated [20].

Systemic Management

Treatment decisions are largely based on expert opinion and the severity of systemic involvement [10]. Immunosuppressive agents, targeted aggressive therapy, and symptomatic management are considered in the literature. Systemic immunomodulators are frequently used, with hydroxychloroquine, corticosteroids, methotrexate, rituximab, and mycophenolate mofetil (MMF) being commonly prescribed [10,23]. Similar to adult practice, stronger immunosuppressive agents such as rituximab and cyclophosphamide are reserved for severe or organ-threatening systemic complications, such as mucosa-associated lymphoid tissue lymphoma, severe renal disease, pulmonary hypertension, and neurological involvement [10]. As an example, a serious manifestation, such as diffuse alveolar hemorrhage, should be managed with high-dose corticosteroids and intravenous rituximab [22]. Topical interventions are suggested as symptomatic agents to provide relief from dryness. They are crucial to diagnosis and symptomatic treatment at an early age to prevent the recurrence of complications in the course of time, including corneal melting [24]. In conclusion, the identified differences in the pathophysiology and clinical/diagnostic settings necessitate the application of a multidisciplinary approach encompassing such clinical specialists and diverse professionals as pediatric rheumatologists, ophthalmologists, and oral health professionals aimed at, first and foremost, tracking the manifestations of inflammation and systemic activity at an earlier stage than any other examination that helps reveal a glandular dysfunction [18]. Further, it is important to continuously monitor due to the high rate of systemic activity at the onset and because of the risk of the additional development of other serious diseases [19].

Diagnostic challenges posed by similarities to other rheumatic conditions

Juvenile Idiopathic Arthritis and Joint Involvement

Among the most significant challenges with cSjD differentiation is the tendency of the disease to be characterized by musculoskeletal manifestations that resemble juvenile idiopathic arthritis and other arthropathies. Prominent joint symptoms, including arthritis and/or arthralgia, are mentioned as one of the most common extracellular manifestations of cSjD in children. In one large cohort, joint involvement was the most reported extracellular manifestation (89 out of 110 cases analyzed) [13]. In an international cohort of 300 children with SD, arthralgia was the most common non-sicca symptom (54%) [8]. Juvenile idiopathic arthritis itself is reported as the most commonly associated autoimmune disease in published cSjD cases (18 cases identified in a literature review) [13]. In non-specific arthritis, children may present with chronic arthritis symptoms that do not meet the International League of Associations for Rheumatology (ILAR) classification criteria for JIA, but still warrant evaluation for cSjD [18,21].

Systemic Lupus Erythematosus and Lupus-Like Phenotypes

cSjD shares substantial clinical and serological overlap with SLE, making precise differentiation difficult, particularly early in the disease course. Both SLE and cSjD are complex autoimmune conditions exhibiting chronicity and autoinflammation, leading to the historical consideration that SS was a benign form of SLE. Serological tests frequently overlap, as anti-SSA and anti-SSB antibodies are found at similar rates in SLE associated with SD (SLE + SD) [25]. cSjD can present with lupus-like features, such as rash or purpura (observed in 51.3% of one Chinese pediatric cohort) [19]. The appearance of photosensitive erythema or cutaneous vasculitis in children may prompt consideration of cSjD alongside SLE [12,18]. Although difficult, the presence of specific markers, such as anti-dsDNA antibodies, antiphospholipid antibodies, and reduced complement levels (C3, C4, CH50), should primarily suggest the diagnosis of SLE, helping distinguish it from cSjD [25].

Other Inflammatory and Infectious Conditions

The characteristic presentation of cSjD often overlaps with conditions typically managed outside of rheumatology, leading to diagnostic confusion. Juvenile recurrent parotitis, recurrent or persistent parotitis (glandular enlargement), is the most common presenting symptom in cSjD, occurring in 47% to 75% of pediatric cases [11,23]. However, juvenile recurrent parotitis is a common differential diagnosis for this presentation. Differentiating SD from juvenile recurrent parotitis is challenging because up to 25% of children with SD may lack SD-specific autoantibodies. The suspicion exists that juvenile recurrent parotitis may represent an early stage or incomplete form of SD that may later progress [11].

The differential diagnosis for recurrent or persistent salivary gland enlargement must exclude infections (viral, bacterial, tuberculosis) and benign or malignant tumors (e.g., Burkitt lymphoma) [18]. A high index of suspicion is required to perform a specific workup, as parotitis may be misleading given its wide differential diagnosis in pediatrics. However, other systemic autoimmune diseases, such as juvenile dermatomyositis and mixed connective tissue disease, have also been reported in association with cSjD [13].

Case Studies and Clinical Examples of Misdiagnosis or Late Diagnosis

The complexity of cSjD often results in a significant delay between the onset of the first clinical manifestation and a definitive diagnosis. In one Italian cohort, the mean time to diagnosis was 1.8 years [13].

Case study 1: Progression to systemic lupus erythematosus/overlap syndrome: A case reported an adolescent girl who was initially diagnosed with SD and followed for six years based on symptoms such as parotid swelling, cyanosis in fingers, leg pain, and positive anti-SSA/SSB and ANA [25]. Six years later, she developed hypocomplementemia (reduced C3 and C4), significantly increased anti-dsDNA, massive proteinuria (7 g/m²/day), and was subsequently diagnosed with SLE nephritis via a kidney biopsy. In this case, an analysis of how cSjD can develop into or result in an overlap syndrome of SLE can be seen, where the first classification is incredibly three-dimensional [25]. Three Chinese patients, originally diagnosed with cSjD, developed SLE over time, thus requiring close attention to this development over time [19].

Case study 2: Misdiagnosis as pulmonary or airway disease: Due to the non-specific nature of systemic manifestations, cSjD symptoms may be misattributed to more common pediatric conditions: asthma/respiratory misdiagnosis and idiopathic pulmonary hemosiderosis. A male pediatric patient with cSjD presented with significant respiratory symptoms at an early age that were initially diagnosed as asthma based on symptoms and obstructive pulmonary function tests. The SD diagnosis was delayed by six years until worsening, refractory respiratory symptoms, and recurrent parotitis prompted a rheumatological evaluation [16]. Idiopathic pulmonary hemosiderosis, the life-threatening manifestation of diffuse alveolar hemorrhage, a rare presentation of cSjD, has been reported. Historically, patients with recurrent diffuse alveolar hemorrhage in childhood may be diagnosed with idiopathic pulmonary hemosiderosis, an unidentifiable disease. Researchers suggest that cSjD may have been overlooked as the underlying cause of alveolar hemorrhage in some idiopathic pulmonary hemosiderosis cases [22].

Case study 3: Misdiagnosis and late diagnosis of lymphoma: The similarity to lymphoid malignancies may result in misdiagnosis or late diagnosis of SD. In two children who developed EMZL in relation to SD, the fine-needle file study of the salivary glands showed non-specific findings, which added to the late diagnosis of SD. This is one of the pitfalls in diagnosis, which can be used only when referring to initial findings in glands, and these findings need to be separated as those of parotitis or malignancy [15]. Characteristics of cSjD, including younger age of diagnosis and foveal swelling, are risk factors of malignancy in adults. Pediatric rheumatologists state that the cases of mucosa-associated lymphoid tissue lymphoma related to cSjD are being treated, with one of the cases ending in death, which highlights the importance of the severe nature of the given potential complication and the necessity of constant control [23].

Case study 4: Uncharacteristic extraglandular presentation: In cases where cSjD is not accompanied by the characteristic glandular swelling or the sicca symptoms, the diagnosis is very challenging, and at times broad differential diagnoses are required: In a single study group, 27 patients with extraglandular manifestations, specifically joint and CNS, were not found to have SS-specific manifestations such as parotitis or sicca symptoms on first presentation [13]. A teenage patient with the beginning of her psychosis expressed it as the first sign of SSS [18], which shows that neurological involvement may be the initial and non-informative symptom.

Long-term outcomes of cSJD

The long-term consequences of cSjD are very complicated and usually not well studied because of the rarity of the disease and the unavailability of strong and prolonged prospective research. Nevertheless, there are indications available that a chronic and systemic course is observed, in which active attention must be paid to the development of dangerous organopathy and the development of additional autoimmune diseases [3,16].

Long-term Effect on Quality of Life, Growth, and Development

SD in childhood affects the quality of life and development of a child negatively, with chronic symptoms and dental problems. Fatigue, dry eyes, parotitis, dry mouth, and pain are the symptoms cited by pediatric rheumatologists as the most common that affect cSjD patients’ quality of life. Commencing local therapy early, including artificial tears and eyelid care, is important to enhance the quality of life and avoid serious side effects in the long term, including corneal melting. Xerostomia is associated with high levels of morbidity, particularly in relation to the condition of the dental system, because of hyposalivation and changes in oral microbiota. Several cases of dental caries have been reported. Diagnosis of dental diseases and hygiene are important at early stages to preserve oral performance. Physical development can be impacted by systemic disease development in childhood. An SD patient who was followed and subsequently developed SLE was observed to have a body weight and height that were below the third percentile. cSjD have been reported to have retarded growth [3,4,16,17,24].

Psychological Effects of Childhood Chronic Autoimmune Disease

Cerebral syndrome of multiple sclerosis disseminated (cSjD) may be connected with severe psychological and behavioral problems due to its chronic and systemic nature. As primary psychiatric disorders, neurological involvement may manifest itself. Patients with CNS involvement have been described as having psychiatric disorders, anxiety, psychotic manifestations, conversion disorder, and behavior disorders. Using rituximab was effective in the treatment of SS with psychiatric symptoms among adolescents, with a significant improvement and antipsychotic discontinuation. Subjective symptoms in the form of fatigue and pain are enumerated as some of the most common clinical symptoms that have an impact on the quality of life, highlighting the mental and physical pressure of living with a permanent autoimmune disease daily. There is no clinical diagnostic standardization in children, and there is a heterogeneous presentation, which leads to delayed diagnosis (an average of 1.8 years in one study), which can cause long-term stress to the child and their family [14,17,18,23].

Gaps in present knowledge

The research on cSjD is characterized by numerous critical gaps in knowledge, which will require particular future research to optimize diagnostic procedures and know the natural history, and provide an evidence-based approach to management. The knowledge of natural history and developmental research is required to establish the clinical prognosis of cSjD in the long term [23]. As an example, it is the long-term follow-up studies of individuals who were diagnosed at an early age that would reveal whether patients will ultimately have a complete SD phenotype in their adult life. These would also be beneficial in defining the prognosis of cSjD [6]. It is unclear whether juvenile recurrent parotitis is an autonomous entity or an undeveloped, initial stage of SD that develops further on. Finally, to establish the actual prevalence of complete symptom remission versus the development of typical SD characteristics, studies are needed with a long-term follow-up [11]. No good-quality studies are currently available in cSjD to permit clinical recommendations on the selection of treatment, and research should be conducted to guide therapeutic recommendations in this rare disease [10]. Future research should be able to gather longitudinal data to take a closer look at the natural history of the disease. It is unknown whether inflammatory changes observed in minor salivary gland biopsies (non-focus lymphocytic aggregates) are an early pathology stage because projections of comparable individuals followed up in subsequent biopsies to assess any progressive changes have not been documented. Objective measurements, including salivary gland ultrasonography, will also require longitudinal monitoring of changes with time [11,20].

Need for Biomarkers Beneficial for Early Diagnosis

The subjective nature of cSjD symptoms, the failure of adult criteria, and the difficulty of performing objective tests in children underscore the urgent need for reliable biomarkers for early detection [21]. The utility of several common diagnostic tests is hampered by the lack of pediatric-specific normative values [8,12,20,24]. Formal studies are needed to define child-specific normal ranges for tests such as the Schirmer test and unstimulated whole salivary flow rate [11,20]. Non-invasive diagnostics are a critical need to define the best set of criteria items for classification [8]. Researchers are exploring the use of imaging, such as salivary gland ultrasonography and ultra-high-frequency ultrasound of labial glands, as potential non-invasive methods to aid diagnosis, but further studies are needed to clarify how specific these changes are for SS in children and what threshold yields sufficient validity to establish diagnosis without biopsy. The presence of autoantibodies suggests the autoimmune process is active years before clinical onset. Identifying highly sensitive biomarkers and immunological features reliably detectable early in the disease course is necessary to improve diagnostic sensitivity [8]. Studies should also focus on identifying noninvasive biomarkers to enhance early detection and monitoring of disease activity [24]. Data-driven approaches, such as the development of the Florida Scoring System (FSS) using machine and causal graph learning on prospective pediatric cohorts, aim to identify essential variables and propose a pediatrician-friendly system to assist clinical reasoning and disease monitoring among suspected patients, but the FSS requires validation in large prospective pediatric cohorts [21].

Potential Areas for Research

Genetic studies, epidemiological data, and multicenter studies are needed to overcome the limitations of small, single-center, retrospective studies and collaborative studies, necessitating large-scale research initiatives [8,13,21]. Multicenter collaborative efforts are needed to develop pediatric-specific diagnostic and treatment guidelines for this rare condition [10,20]. The successful use of large international registries, such as the Sjögren Big Data Registry and the International Childhood Sjögren Syndrome Workgroup, demonstrates the possibility of obtaining robust, generalizable datasets for this rare disease [6,8]. The prevalence of cSjD is poorly defined, with an unknown prevalence [16]. Further large-scale international collaborative studies are necessary to better define and understand the natural history of SS in children [6,19]. Although the presence of autoimmunity in the family is a known feature, specific genetic factors influencing cSjD susceptibility and phenotype require further investigation. Future studies should include a larger patient sample and comparison with healthy children to advance understanding [12]. Criteria validation to establish pediatric-specific diagnostic criteria is essential for use in future research studies and for clinical diagnosis [8,10,20]. Potential criteria items need to be considered and evaluated prospectively to determine the optimal set of criteria for children [8,18].

## Conclusions

cSjD is a rare and poorly understood systemic autoimmune illness with a peculiar clinical manifestation. This synthesis reviews the literature and finds a diverse illness profile with recurring parotitis and extraglandular signs as opposed to adult sicca symptoms (dry eyes and mouth). Pediatric rheumatology faces challenges with diagnostic criteria created in adults, such as the 2016 ACR/EULAR criteria, which have low sensitivity in children and delay diagnosis. Research shows that a considerable percentage of children diagnosed with cSjD by specialists do not meet the guidelines. Children show frequent parotitis, arthralgia, lymphadenopathy, and hematological, renal, hepatic, and CNS dysfunction. The review also highlighted the constitutional symptoms such as rash, fever, and joint pain (arthralgia), which are common and often present alongside glandular inflammation. Neurological involvement, including psychiatric symptoms, may also occur, further complicating diagnosis and management. The rare life-threatening consequences include interstitial lung disease, diffuse alveolar hemorrhage, and B-cell lymphoma. Existing studies use non-invasive methods and innovative classification techniques to close the diagnosis gap. New methods for measuring gland inflammation include salivary gland ultrasonography and ultra-high-frequency ultrasonography. To diagnose cSjD more accurately and consistently, empirical approaches such as the Florida Scoring System (FSS) and systematic clinical models such as the three-pathway diagnostic algorithm have been developed. Research is needed to prove diagnostic algorithms. Managing cSjD is highly individualistic and usually based on adult practice. Mild symptoms are treated with non-steroidal anti-inflammatory drugs and hydroxychloroquine, while moderate or severe systemic disease and consequences are managed with corticosteroids, traditional disease-modifying antirheumatic drugs, and biologics such as rituximab. These treatment strategies are devised from low-quality evidence, which is based on case reports and case series, highlighting the need for collaboration, high-quality research, pediatric-specific diagnostic criteria, and further updated guidelines.
